# Absceso parafaríngeo por cuerpo extraño en pediatría: Presentación de caso clínico y revisión de la literatura

**DOI:** 10.23938/ASSN.1172

**Published:** 2026-08-14

**Authors:** Beatriz Heras Cazorla, Jorge Bedia García, Irene Gómez Gregoris, Miguel Víctor Grijalba Uche, Pablo Crespo Escudero

**Affiliations:** Sanidad de Castilla y León (SACYL) Hospital Universitario de Burgos Servicio de Otorrinolaringología Burgos Castilla y León España

**Keywords:** Absceso, Espacio parafaríngeo, Pediatría, Reacción a cuerpo extraño, Caso clínico, Abscess, Parapharyngeal Space, Pediatrics, Foreign-body Reaction, Clinical Report

## Abstract

Las infecciones cervicales profundas en pediatría presentan una incidencia creciente y pueden originar complicaciones graves debido a su clínica inicialmente inespecífica. Se describe el caso de una paciente pediátrica de 12 meses que desarrolló un absceso parafaríngeo con extensión mediastínica tras la ingestión accidental de una espina de pescado, que precisó manejo combinado quirúrgico y conservador con evolución favorable.

El objetivo de presentar este caso clínico representativo de esta patología es revisar la clínica, exploración física, métodos diagnósticos, tratamiento, evolución y seguimiento de un absceso parafaríngeo. Asimismo, mediante una revisión de la literatura publicada se pretende comparar distintas estrategias diagnósticas y terapéuticas y contrastarlas con nuestra práctica clínica, con el fin de aportar apoyo práctico para el manejo de casos similares.

## Abstract (en)

Deep neck infections in pediatric patients are increasingly frequent and may lead to severe complications due to nonspecific early symptoms. We report the case of a 12-month-old female infant who developed a parapharyngeal abscess with mediastinal extension following accidental fish bone ingestion, requiring combined surgical and conservative management, with favorable outcome.

The primary aim of this article is to present a representative clinical case describing the clinical presentation, physical examination, diagnostic assessment, treatment, clinical course, and follow-up of this condition. Additionally, we reviews the available literature to compare different diagnostic and therapeutic approaches and contrast them with our clinical practice, providing practical guidance to support the management of similar cases in the future.

## INTRODUCCIÓN

Las infecciones cervicales profundas son procesos infecciosos de diversa etiología que comprometen los espacios comprendidos entre las distintas fascias del cuello, siendo los espacios parafaríngeos uno de los principales sitios de afectación^1^. Estas infecciones son más frecuentes en población pediátrica, y su incidencia parece estar aumentando^2^. La inespecificidad de la sintomatología inicial (con fiebre como única manifestación clínica), favorece el retraso diagnóstico y, por consiguiente, la aparición de complicaciones graves y el aumento de la morbimortalidad asociada^3^.

Presentamos el caso de una niña de doce meses con un absceso parafaríngeo secundario a cuerpo extraño, con el objetivo de aportar información de utilidad sobre el diagnóstico diferencial, las pruebas complementarias y el tratamiento de esta entidad. Con ello se pretende facilitar su reconocimiento y favorecer un diagnóstico precoz que permita instaurar el adecuado abordaje terapéutico.

## CASO CLÍNICO

Se presenta el caso de una paciente pediátrica de doce meses que acudió al servicio de urgencias hospitalarias por episodio de atragantamiento, mientras ingería pescado, en contexto de un cuadro catarral de aproximadamente diez días de evolución. Fue valorada en su centro de salud el mismo día del episodio de atragantamiento, sin detectarse ningún cuerpo extraño.

**Figura 1:**
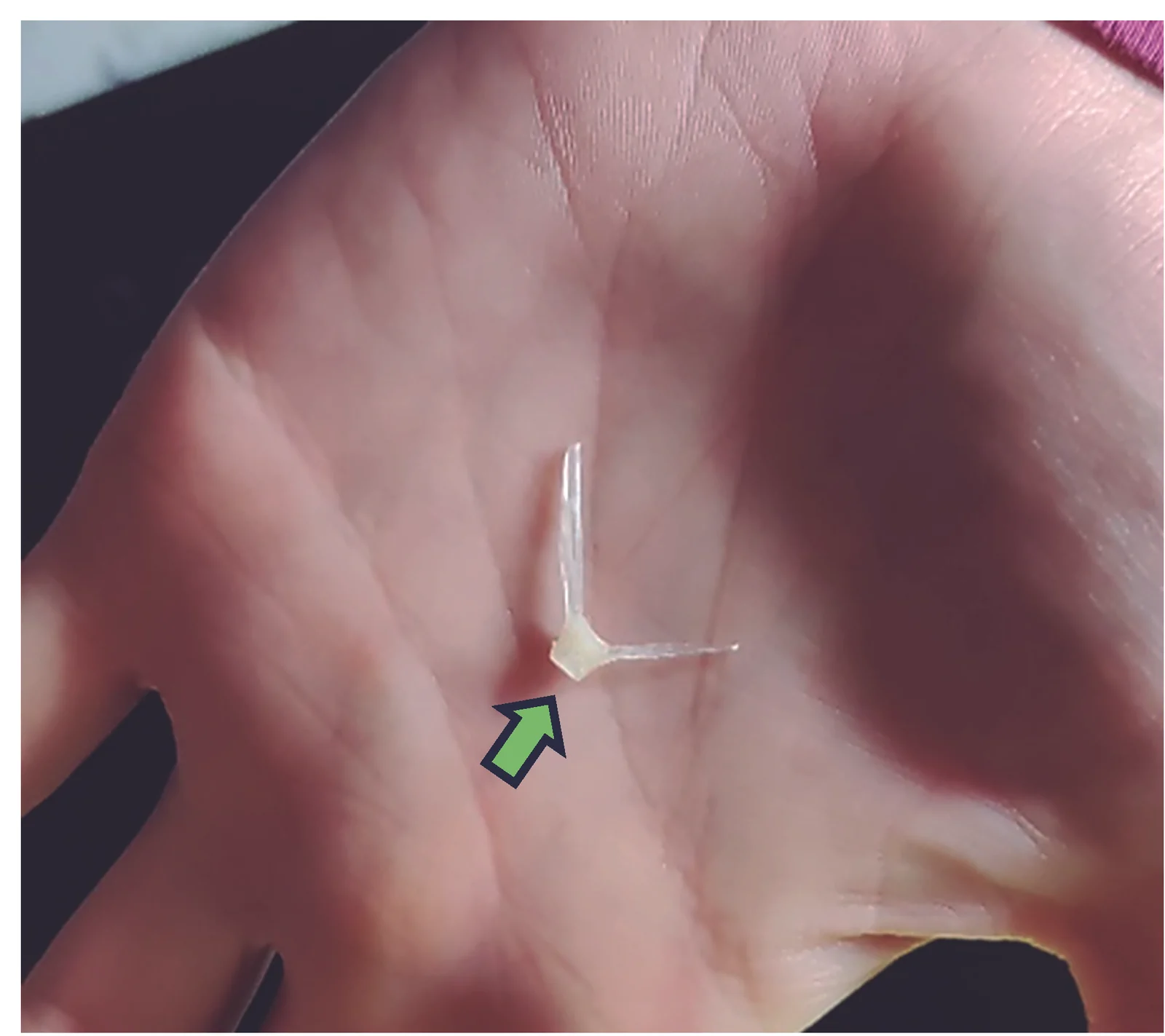
Cuerpo extraño (espina de pescado) expulsada por la paciente durante un acceso de tos.

Al día siguiente comenzó con fiebre de hasta 39 °C de difícil control con tratamiento antipirético, por lo que fue reevaluada 48h después del episodio por su pediatra, nuevamente sin evidencias de cuerpo extraño, aunque presentando hiperemia faríngea y sialorrea. Aproximadamente 48 horas después de esta última valoración, y coincidiendo con un acceso de tos, la paciente expulsó espontáneamente una espina de pescado de gran tamaño (Fig. 1).

Durante los días posteriores los progenitores refirieron persistencia de la odinofagia, la fiebre y el decaimiento, asociados a movilidad cervical limitada y una disminución importante de la ingesta.

**Figura 2:**
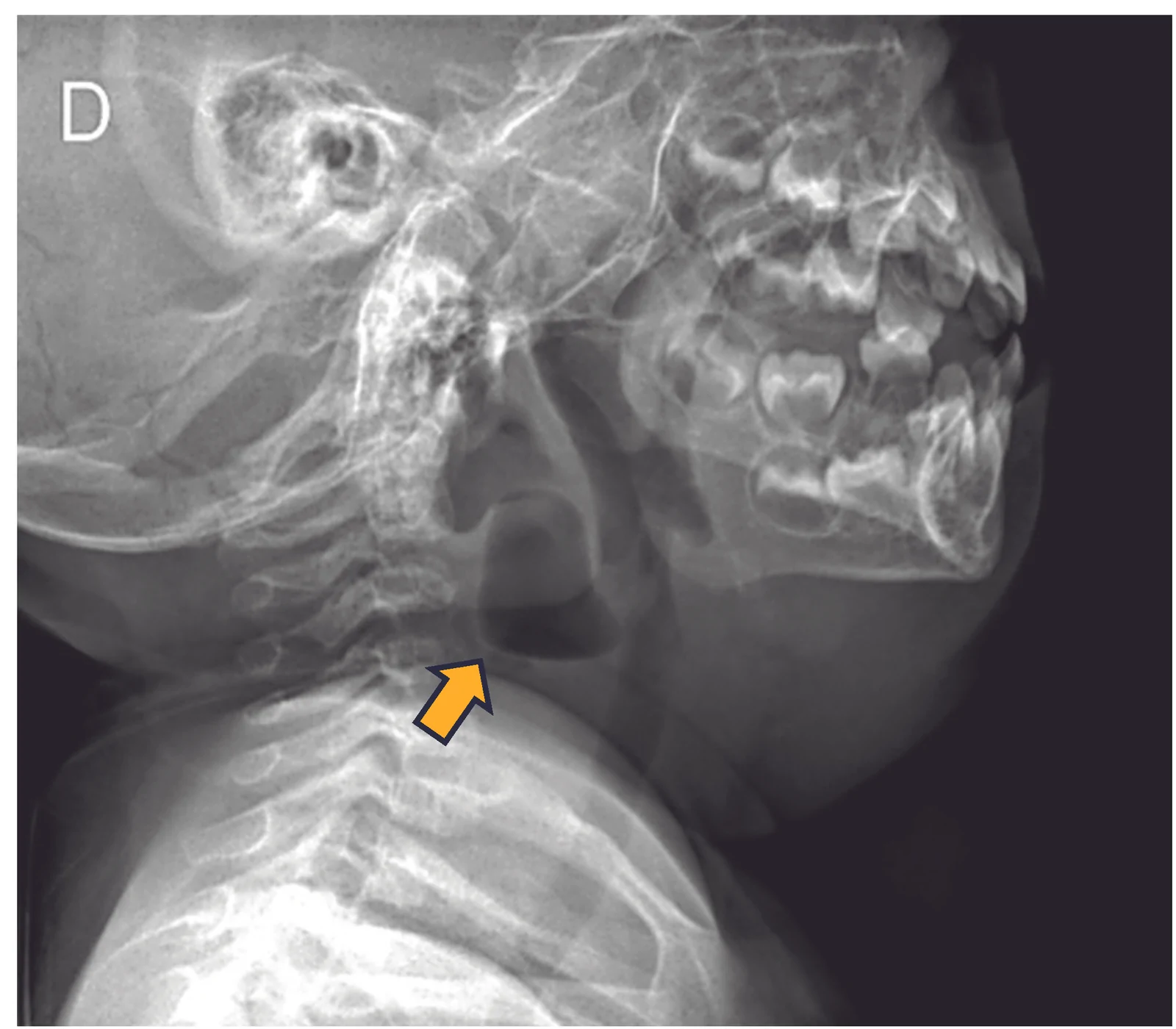
Radiografía cervical lateral en la que se visualiza burbuja de aire (flecha) compatible con absceso cervical profundo.

A los diez días del inicio del cuadro fue valorada en urgencias de su centro de referencia. Durante la exploración se observó empeoramiento clínico (persistencia de fiebre, rechazo de la ingesta, signos de deshidratación y progresivo decaimiento a nivel neurológico) y analítico (leucocitosis con desviación izquierda, y elevación de PCR hasta 78 mg/L). En la radiografía cervical lateral se detectó ensanchamiento retrofaríngeo y la presencia de dos bolsas aéreas (Fig. 2). Por ello, se decidió su derivación urgente a un hospital terciario con disponibilidad de cuidados intensivos pediátricos y cirugía otorrinolaringológica, para valoración y tratamiento especializados.

**Figura 3:**
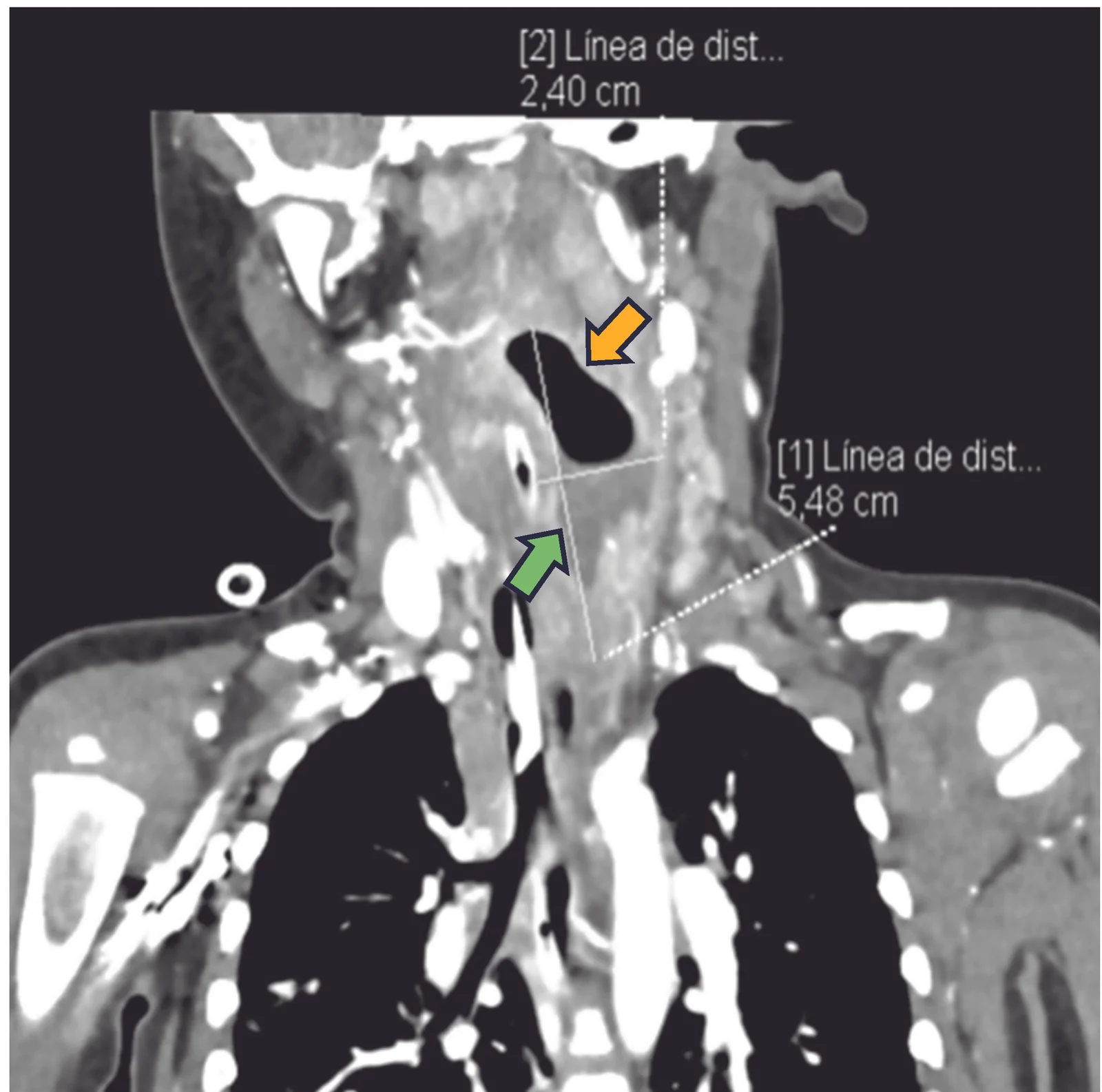
Tomografía axial computarizada (TAC) cervical urgente prequirúrgica. Corte coronal. Se observa colección cervical (flecha verde) y presencia de aire asociada (flecha amarilla).

A su llegada, se contactó con la unidad de cuidados intensivos (UCI) pediátrica y se decidió realizar tomografía axial computarizada (TAC) cérvico-torácica urgente con sedación. Durante la sedación, la paciente se desestabilizó requiriendo intubación urgente. Tras estabilizarla, se realizó la TAC en la que se visualizó un extenso absceso parafaríngeo izquierdo que se extendía desde el suelo de la boca hasta el mediastino superior, con importantes cambios inflamatorios y efecto de masa sobre la vía aérea, que se encontraba desplazada a la derecha (Fig. 3).

Se realizó una intervención quirúrgica urgente para realizar drenaje cervical del absceso parafaríngeo, con salida de abundante contenido purulento que se envió al servicio de Microbiología. En el cultivo se observó crecimiento de *Streptococcus constellatus,* sensible, y *Lactobacillus casei,* resistente únicamente a vancomicina.

Tras cinco días de ingreso en UCI pediátrica y tratamiento antibiótico intravenoso con cefotaxima 200 mg/kg/día y clindamicina 40 mg/kg/día, la paciente fue dada de alta a la planta de hospitalización de Otorrinolaringología, donde continuó el tratamiento antibiótico. La paciente experimentó buena evolución clínica por lo que fue dada de alta a domicilio tras dos días de ingreso, cumpliendo así siete días de tratamiento antibiótico intravenoso.

Once días después del alta hospitalaria, acudió de nuevo a urgencias por varios episodios de fiebre de hasta 38,5 ºC. Se le realizó una resonancia magnética (RNM) cervical en la que se visualizó persistencia de la colección parafaríngea izquierda, con diámetros aproximados de 16x10x41 mm (transversal, antero-posterior, cráneo-caudal) con extensos cambios inflamatorios y efecto de masa sobre la vía aérea a nivel de la orofaringe, con colapso prácticamente completo del seno piriforme izquierdo, así como múltiples adenopatías reactivas en todos los niveles cervicales, las mayores de 17x11 mm a nivel IIB izquierdo.

Se decidió ingresarla de nuevo en la planta de hospitalización de Otorrinolaringología, con tratamiento antibiótico intravenoso (amoxicilina/clavulánico 100 mg/kg/día). Durante el ingreso, radiología intervencionista realizó punción-aspiración de la colección parafaríngea izquierda. En el cultivo de la muestra extraída se aisló *Veillonella atypica*, resistente únicamente a penicilina.

Se repitió prueba de imagen (RNM) tras catorce días de ingreso en planta con tratamiento antibiótico intravenoso dirigido, apreciándose una importante mejoría radiológica, con mínimos cambios flemonosos y ausencia de abscesos asociados, por lo que es dada de alta a domicilio.

Un mes después del alta hospitalaria, la niña fue valorada de nuevo en consultas externas de Otorrinolaringología, apreciándose buen aspecto de la herida quirúrgica, con palpación cervical sin alteraciones y nasofibrolaringoscopia dentro de la normalidad, por lo que fue dada de alta de nuestro servicio para continuar seguimiento por su pediatra.

## DISCUSIÓN

Las fascias cervicales están estructuradas de tal forma que dividen el cuello una serie de espacios cervicales supra e infrahioideos. Dentro de los suprahioideos se encuentra el espacio parafaríngeo, que se extiende desde base de cráneo hasta el hioides, limitado anteriormente por rafe pterigomandibular y posteriormente por fascia prevertebral. La musculatura estiloidea y la apófisis estiloides dividen el espacio parafaríngeo en otros dos: el preestileo, que contiene la arteria maxilar interna y el nervio lingual, y el retroestileo, que contiene la carótida interna, la yugular interna, los pares craneales IX, X, XI y XII, y el ganglio simpático cervical superior^4^^,^^5^.

La etiología más frecuente de las infecciones del espacio preestileo son las infecciones dentales seguidas de los flemones periamigdalinos -más frecuentemente- y de las faringoamigdalitis. Sin embargo, las infecciones retroestileas suelen producirse como complicación de una infección preestiloidea o retrofaríngea previa (flemón periamigdalino, infecciones dentales, angina de Ludwig o heridas mucosas secundarias a cuerpos extraños). Este último mecanismo etiopatogénico es poco frecuente en la edad pediátrica, especialmente en lactantes^6^ y, en el caso presentado, la perforación mucosa por una espina de pescado parece constituir el desencadenante del proceso infeccioso.

En términos epidemiológicos, los abscesos parafaríngeos y retrofaríngeos representan entidades poco frecuentes en la infancia, aunque se ha observado un incremento de su incidencia en las últimas décadas^7^. La mayoría de los casos se concentran en menores de seis años, probablemente en relación con la mayor frecuencia de infecciones respiratorias altas y con la persistencia de tejido linfático parafaríngeo y retrofaríngeo^8^. Más concretamente, en menores de seis años predominan las infecciones del espacio retrofaríngeo, que representan hasta el 70% de los casos en menores de cuatro años, mientras que en niños mayores de seis años, el absceso parafaríngeo constituye el 61,5% de las infecciones cervicales^9^.

Las manifestaciones clínicas de los abscesos parafaríngeos son variadas y, en ocasiones, inespecíficas, como en el caso presentado. En la afectación del espacio preestileo, lo más frecuente es la aparición de fiebre con odinofagia, trismus e hipersalivación, pudiendo producirse un abombamiento de la pared faríngea lateral, con amígdala y úvula desplazadas hacia la línea media y tumefacción cervical dolorosa parotídea o submandibular. Sin embargo, cuando el espacio retroestíleo se encuentra afectado, predominan la fiebre, la odinofagia, la rigidez cervical, la tumefacción cervical y la tumefacción lateral retroamigdalina^10^. Nuestra paciente presentó varios de estos hallazgos, incluyendo una disminución importante de la ingesta. Sin embargo, la inespecificidad de los síntomas iniciales y la expulsión espontánea de la espina de pescado a las 48 horas del episodio pudieron contribuir al retraso diagnóstico, ya que la persistencia de la sintomatología fue interpretada inicialmente como parte de la evolución del proceso inflamatorio local.

La afectación de este espacio puede conllevar complicaciones como la tromboflebitis de la vena yugular interna (síndrome de Lemierre), la difusión al espacio retrofaríngeo con obstrucción de la vía aérea o aparición de neumonías aspirativas e, incluso, la extensión torácica, pudiendo provocar mediastinitis o pericarditis con riesgo de taponamiento cardíaco y evolucionar, en los casos más graves, hacia un shock séptico^11^. Nuestro caso ejemplifica la gravedad potencial de estas infecciones, ya que la TAC evidenció una extensa colección parafaríngea con un importante efecto de masa sobre la vía aérea. Además, la desestabilización clínica durante la sedación pone de manifiesto el elevado riesgo de compromiso respiratorio asociado a estos procesos y la necesidad de realizar una valoración precoz y multidisciplinar, tal y como se ha descrito previamente en la literatura pediátrica^10^^,^^11^.

Por ello, en estas infecciones es fundamental establecer un diagnóstico precoz que permita iniciar el tratamiento oportuno y evitar la aparición de complicaciones graves. Para confirmar la presencia de un absceso cervical cuando la anamnesis y la exploración física orientan a ello, es necesario realizar una prueba de imagen. En nuestro caso, la radiografía cervical lateral realizada inicialmente mostró hallazgos sugestivos de infección profunda cervical que motivaron la derivación urgente a un centro terciario. Posteriormente, la TAC cérvico-torácica permitió definir con exactitud la extensión del absceso y orientar la actitud terapéutica. Según Ruiz de la Cuesta y col^12^ en su estudio sobre abscesos cervicales profundos pediátricos realizado a lo largo de quince años, las más empleadas son la TAC en abscesos profundos y la ecografía en colecciones superficiales. La RNM se está empleando con mayor frecuencia tanto como herramienta diagnóstica como para el control radiológico ante la sospecha de persistencia de un absceso cervical profundo tras la cirugía, evitando así la exposición a radiación ionizante^8^. En nuestra paciente se realizó inicialmente una TAC urgente, dada la situación de inestabilidad clínica y la sospecha de una complicación grave; sin embargo, en un contexto de estabilidad clínica y ante la necesidad de reevaluación evolutiva durante el segundo ingreso, se optó por la realización de RNM cervical, minimizando así la exposición acumulada a radiación.

En los abscesos cervicales profundos pediátricos es importante iniciar la administración temprana de antibioterapia empírica de amplio espectro (amoxicilina/ácido clavulánico o ampicilina/sulbactam)^8^ antes de continuar con la antibioterapia dirigida según los resultados del cultivo microbiológico. En nuestro caso, el aislamiento inicial de *S.constellatus* -caracterizado por su elevada capacidad para la formación de abscesos- junto con *L. casei*, constituyó un hallazgo poco habitual, así como el aislamiento posterior de *V. atypica*, anaerobio comensal de la cavidad oral raramente implicado en infecciones profundas cervicales. Estos hallazgos apoyan la naturaleza polimicrobiana de estas infecciones, predominando estreptococos y anaerobios de la flora orofaríngea, y ponen de relieve la importancia de obtener cultivos microbiológicos para adecuar el tratamiento antibiótico^13^.

La administración empírica de cefotaxima y clindamicina durante el primer ingreso se asoció a una mejoría clínica significativa y al control de la infección, hallazgos que posteriormente fueron corroborados por el antibiograma, que evidenció sensibilidad del microorganismo aislado frente a ambos antibióticos. Según la revisión de Esposito y col, también las pautas antibióticas basadas en la combinación de un antibiótico de amplio espectro con clindamicina o metronidazol han demostrado ser una opción terapéutica eficaz frente a los microorganismos aerobios y anaerobios habitualmente implicados en los abscesos cervicales profundos^8^, como en el caso de nuestra paciente.

En niños mayores de cuatro años con abscesos retrofaríngeos o parafaríngeos menores de 2,5-3 cm en la TAC, sin complicaciones asociadas y con una respuesta clínica favorable durante las primeras 48-72 horas, puede optarse por un manejo únicamente conservador mediante antibioterapia intravenosa, evitando la intervención quirúrgica^2^^,^^8^. Nuestra paciente no cumplía dichos requisitos (compromiso de vía aérea y edad menor de cuatro años), por lo que se decidió abordaje quirúrgico para drenar la colección parafaríngea. Sin embargo, durante el segundo ingreso, y pese a la persistencia de una colección residual, el menor tamaño de esta y la ausencia de nuevas complicaciones permitieron un abordaje más conservador mediante antibioterapia intravenosa dirigida y punción-aspiración guiada por radiología intervencionista, con evolución clínica y radiológica favorable. Este hecho pone de manifiesto que el tratamiento debe individualizarse en función de la situación clínica del paciente y de la evolución radiológica del proceso infeccioso.

En conclusión, los abscesos parafaríngeos constituyen una patología potencialmente grave cuya presentación clínica puede ser inespecífica, dificultando el diagnóstico en fases iniciales. Una anamnesis exhaustiva y una exploración física dirigida son esenciales para su reconocimiento precoz, permitiendo instaurar un tratamiento oportuno y minimizar el riesgo de complicaciones, especialmente el compromiso de la vía aérea y la diseminación de la infección.

## Data Availability

Los datos que respaldan los hallazgos de este estudio están incluidos en el artículo. Debido a la naturaleza clínica del caso y a la necesidad de preservar la confidencialidad del paciente, no se dispone de bases de datos adicionales de acceso público. Información adicional podrá ser facilitada por el autor de correspondencia previa solicitud razonable y conforme a las normativas éticas y de protección de datos vigentes.
